# SETDB2 interacts with BUBR1 to induce accurate chromosome segregation independently of its histone methyltransferase activity

**DOI:** 10.1002/2211-5463.13761

**Published:** 2024-01-09

**Authors:** Yanhong Tu, Haomiao Zhang, Jialin Xia, Yu Zhao, Ruifang Yang, Jing Feng, Xueyun Ma, Jing Li

**Affiliations:** ^1^ School of Laboratory Medicine and Biotechnology Southern Medical University Guangzhou China; ^2^ The Second Affiliated Hospital The Chinese University of Hong Kong Shenzhen China; ^3^ The Third School of Clinical Medicine Southern Medical University Guangzhou China; ^4^ Anhui University of Science and Technology Affiliated Fengxian Hospital Shanghai China; ^5^ Shanghai Key Laboratory of Regulatory Biology Institute of Biomedical Sciences and School of Life Sciences East China Normal University Shanghai China

**Keywords:** APC/C, BUBR1, CDC20, chromosome segregation, mitosis, SETDB2

## Abstract

SETDB2 is a H3K9 histone methyltransferase required for accurate chromosome segregation. Its H3K9 histone methyltransferase activity was reported to be associated with chromosomes during metaphase. Here, we confirm that SETDB2 is required for mitosis and accurate chromosome segregation. However, these functions are independent of its histone methyltransferase activity. Further analysis showed that SETDB2 can interact with BUBR1, and is required for CDC20 binding to BUBR1 and APC/C complex and CYCLIN B1 degradation. The ability of SETDB2 to regulate the binding of CDC20 to BUBR1 or APC/C complex, and stabilization of CYCLIN B1 are also independent of its histone methyltransferase activity. These results suggest that SETDB2 interacts with BUBR1 to promote binding of CDC20 to BUBR1 and APC3, then degrades CYCLIN B1 to ensure accurate chromosome segregation and mitosis, independently of its histone methyltransferase activity.

## Graphical abstract

The histone methyltransferase SETDB2 interacts with BUBR1 and is required for binding of CDC20 to BUBR1 and APC, thereby mediating degradation of CYCLIN B1 to guarantee accurate chromosome segregation and mitosis exit. This occurs independently of SETDB2's histone methyltransferase activity.

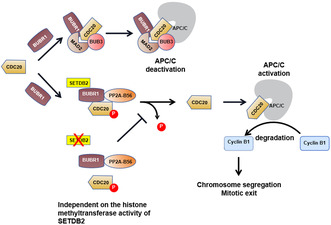

**Abbreviations**
APC/C: anaphase promoting complex/cyclosome
APC3: anaphase promoting complex subunit 3
BUBR1: BUB1 mitotic checkpoint serine/threonine kinase B
CDC20: cell division cycle 20
IP: immunoprecipitation
MCC: mitotic checkpoint complex
NC: negative control
SAC: spindle assembly checkpoint
SD: standard deviation
SETDB2: SET domain bifurcated histone lysine methyltransferase 2

SETDB2 (CLLD8 or KMT1F) as an H3K9 histone methyltransferase [1, 2, 3], plays critical roles in immune system [3, 4, 5] and embryonic development [6, 7]. In cancer research, SETDB2 plays oncogenic roles [8], involved in cell cycle dysregulation [9], cancer stem cell maintenance [10], and metastasis [11]. During mitosis, SETDB2 induces trimethylation of both interspersed repetitive elements and centromere associated repeats, and then recruits heterochromatin protein HP1 to centromeres [2]. Knocking down SETDB2 leads to delayed mitosis and abnormal chromosome segregation [2], which suggests that SETDB2 is required for accurate chromosome segregation. However, the roles and mechanism of SETDB2 in mitosis and chromosome segregation need further study.

The mechanism of chromosome segregation and mitosis regulation are central mysteries of cell division. During mitosis, the anaphase promoting complex/cyclosome (APC/C) is an evolutionally conserved multisubunit E3 ubiquitin ligase that triggers the metaphase to anaphase transition [12]. The exquisite regulation of APC/C guarantees accurate chromosome segregation [13]. In early mitosis, CDC20 is activated, then binds and activates APC/C to form the APC/C^CDC20^ complex [14]. Once all the chromosomes achieve proper microtubule biorientation in metaphase, the APC/C^CDC20^ complex ubiquitinates the anaphase inhibitors SECURIN and CYCLIN B1 to promote sister chromatid separation and mitosis exiting [15, 16]. BUBR1, as a CDC20 interaction protein, is involved in regulating CDC20 during mitosis. On the one hand, BUBR1, CDC20, MAD2, and BUB3 assemble to form the mitotic checkpoint complex (MCC) [17, 18, 19]. The MCC is released to bind and deactivate the APC/C complex, then leads to SAC (sspindle assembly checkpoint)‐dependent mitotic arrest [15, 16]. On the other hand, phosphorylation of CDC20 reduces its binding affinity for APC/C and allows for the accumulation of CYCLIN B1, then leads to the G2‐to‐M transition [20]. BUBR1 acts as a substrate specifier for PP2A‐B56 to efficiently dephosphorylate CDC20 [21], then leads CDC20 to bind and activate the APC/C complex [22]. However, how does BUBR1 regulate CDC20 and APC/C complex activation during mitosis is unclear.

In our study, we found that the functions of SETDB2 on regulating mitosis and chromosome accurate segregation are independent of its histone methyltransferase activity. The mechanism analysis shows that SETDB2 can act as a novel BUBR1 interaction protein, which was required for CDC20 binding to BUBR1 or APC/C complex, then lead to CYCLIN B1 degradation. The functions of SETDB2 on regulating the binding ability of CDC20 with BUBR1 or APC/C complex, and the CYCLIN B1 stabilization, are also independent of its histone methyltransferase activity. These results indicate that histone methyltransferase SETDB2 interacts with BUBR1 to promote CDC20 to bind BUBR1 and APC3, then degrades CYCLIN B1 to guarantee accurate chromosome segregation and mitosis exiting in a histone methyltransferase activity‐ in an independent way.

## Materials and methods

### Plasmids, siRNA, cell lines, and antibodies

The eukaryotic expression plasmids pcDNA3.1‐HA‐SETDB2, pEGFP‐SETDB2‐WT, and prokaryotic expression plasmid pQE31‐His‐SETDB2 were constructed using lentivirus plasmid Plvx‐IRES‐Neo‐FLAG‐SETDB2 (described in Ref. [10]) as a polymerase chain reaction (PCR) template. The sequence of SETDB2 is shown in Table S1. The eukaryotic expression plasmid pEGFP‐SETDB2‐2GA was constructed using the pEGFP‐SETDB2‐WT plasmid as a PCR template follow Site‐Directed Mutagenesis protocol. The sequence of SETDB2‐2GA is shown in Table S2.

The small Interfering RNA negative control (NC) and SETDB2 siRNA (siSETDB2‐1 and siSETDB2‐3) were purchased from GenePharma (Shanghai, China). The sequence of siRNA is shown in Table S4.

The cell lines were purchased from the American Type Culture Collection (ATCC, Gaithersburg, MD, USA); MCF7, HEK293T, and MDA‐MB‐231 cells were cultured with Dulbecc’'s modified Eagle medium (DMEM) with 10% fetal bovine serum (FBS) and 1% penicillin–streptomycin solution (PS), and SUM159PT cells were cultured with Ha’'s F12 with 5% fetal bovine serum (FBS), 10 mm HEPS, 1 μg·mL^−1^ hydrocortisone, 0.005 mg·mL^−1^ insulin, and 1% PS; all cells grew at 37 °C and 5% CO_2_ with saturating humidity.

Antibodies against GAPDH (#AC002; ABclonal Technology, Wuhan, China), CDC20 (#A15656; ABclonal Technology), β‐ACTIN (#20536‐1‐AP; Proteintech, Wuhan, China), MAD2 (#66014‐1‐Ig; Proteintech), MPS1 (#ab11108; Abcam, Cambridge, UK), APC3 (#sc‐9972; Santa Cruz Biotechnology, Dallas, TX, USA), and CYCLIN B1 (#A2056; ABclonal Technology, Wuhan, China) were used for western blot.

Antibodies against SETDB2 (#PA5‐30848; Invitrogen, Carlsbad, CA, USA), CDC20 (#sc‐5296; Santa Cruz Biotechnology), BUBR1 (#11504‐2‐AP; Proteintech), and HA (#51064‐2‐AP; Proteintech) were used for western blot and immunoprecipitation.

An antibody against β‐TUBULIN (#ab6046; Abcam) and H3K9me3 (#pAb‐056‐50; Diagenode, Denville, NJ, USA) was used for immunofluorescence.

### Site‐directed mutagenesis

The pEGFP‐SETDB2‐WT plasmid as a PCR template was used for plasmid pEGFP‐SETDB2‐2GA construction. The mutation sites are shown in Table S2. The primers used for site‐directed mutagenesis are shown in Table S3. Ten nanogram temple plasmid, 125 ng sense primer, 125 ng antisense primer, 5 μL 10 × reaction buffer, 1 μL dNTP mix, and 1 μL *PfuTurbo* DNA polymerase (2.5 U·μL^−1^) were used in a 50‐μL PCR reaction eactim. The cycling parameters for PCR reaction were: 95 °C, 30 s, 1 cycle; 95 °C, 30 s, 52 °C, 1 min, 68 °C, 1 min·kb^−1^ of plasmid length, 18 cycles; 4 °C, 5 min. Then add 1 μL *DpnI* (10 U·μL^−1^) in the reaction to digest the template DNA at 37 °C for 1 h. After digestion, the *DpnI‐*treated DNA was transformed into *Escherichia coli*. The clones of bacteria were sequenced to screen the positive clones. Then the plasmid DNA was extracted for further experiments.

### Cell transfection

The cells were growth to 80% confluence. For siRNA transfection, 100 pmol siRNA and 5 μL Lipofectamine 3000 (#L3000015; Invitrogen) were used per a 3.5‐cm dish. For plasmid transfection, 2 μg DNA, 4 μL Lipofectamine3000, and 4 μL P3000 (#L3000015; Invitrogen) were used per 3.5‐cm dish. We exchanged the flesh medium for the cells after 24 h transfection. The cells were analyzed after 48–72 h transfection.

### Cell synchronized

For immunoprecipitation, MCF7 cells and 293T cells were treated with thymidine (2 mm, #T1895; Sigma‐Aldrich, St. Louis, MO, USA) for 18 h and released into fresh medium for 10 h and then blocked with 2 mm thymidine again for 18 h. After that, the cells were released into fresh medium for 2 h and treated with nocodazole (0.1 μg·mL^−1^, #M1404; Sigma‐Aldrich) for 10 h. Then the mitotic cells were collected for immunoprecipitation.

For analysis of flow cytometry and western blot, SUM159PT cells, MCF7 cells, and 293T cells were treated with nocodazole (0.1 μg·mL^−1^, #M1404; Sigma‐Aldrich) for 10 h and released into nocodazole‐free medium; then the cells were collected at the indicated time for further analysis.

### Cell cycle assay

After synchronizing, the cells were trypsinized and collected by spinning. Then the cell pellets were resuspended in cold 70% ethanol solution for fixation. After being fixed for 12–24 h in 4 °C, the cells were stained with PropidiumIodide (#C1052; Beyotime, Shanghai, China) following the supplier's manual and analyzed with a BD LSRFortessa FACScan flow cytometer and bd facsdiva software (BD Biosciences, Franklin Lakes, NJ, USA).

### Immunofluorescence

Cells were fixed with 4% paraformaldehyde for 20 min followed by a 15‐min cell permeabilization with 1% (vol/vol) Triton X‐100 in PBS. After permeabilization, cells were blocked in PBS with 2% (wt/vol) bovine serum albumin (BSA) for 1 h at 37 °C. Antibody dilutions were performed in 2% BSA and added into the cell culture cluster overnight at 4 °C. After primary antibody incubation, cells were rinsed and incubated for 30 min at 37 °C with secondary antibodies conjugated to Alexa Fluor 568 (#A‐11036; Invitrogen). Then the cells were stained with DAPI (1 ng·mL^−1^). The cells were quickly rinsed in PBS and then imaged and analyzed by olympus cellsens software (Olympus, Tokyo, Japan).

### Immunoprecipitation and pulldown

For immunoprecipitation, the cells were collected by centrifugation and were lysed in lysis buffer (50 mm Tris–HCl, pH 7.4, 200 mm NaCl, 1 mm EDTA, 0.1% Triton X‐100, 8% glycerol, protease inhibitor cocktail [#P1010; Beyotime]) on ice for 30 min. Then the cells were lysed and the cell lysate supernatant was incubated with 1 μg antibodies and 10 μL protein A + G‐agarose beads (#P2055; Beyotime) or 10 μL anti‐HA nanobody conjugated agarose beads (#HNA‐25‐500; NuoyiBio, Tianjin, China) by rotation overnight at 4 °C. After being washed 5 times (10 min for each time) using washing buffer (50 mm Tris–HCl, pH 7.4, 150 mm NaCl, 1 mm EDTA, 0.1% Triton X‐100, protease inhibitor cocktail [#P1010; Beyotime]), the beads were analyzed by western blot.

For the pulldown assay, His‐SETDB2 protein was expressed in *E. coli*. The bacteria pellets were collected and resuspended in lysis buffer (50 mm NaH_2_PO_4_, 300 mm NaCl, 10 mm imidazole, pH 8.0, inhibitor cocktail [#P1010; Beyotime]). After sonication, the supernatant was collected and incubated with the BeyoGold™ His‐tag Purification Resin (#P2210FT; Beyotime) beads overnight at 4 °C. After being washed 5 times (10 min for each time) using washing buffer (50 mm NaH_2_PO_4_, 300 mm NaCl, 20 mm imidazole, pH 8.0), the beads were incubated with MCF7 cell lysate supernatant by rotation overnight at 4 °C for pulldown. Then the beads were washed 5 times (10 min for each time) using pulldown washing buffer (50 mm Tris–HCl, pH 7.4, 150 mm NaCl, 1 mm EDTA, 0.1% Triton X‐100, 20 mm imidazole, protease inhibitor cocktail [#P1010; Beyotime]), and analyzed by western blot.

### Core histone extract

For histone western blot analysis, the protein should be extracted following the core histone extract protocol. In brief, cells in a 10‐cm dish were collected and resuspended in 200 μL cold lysis buffer (20 mm Tris, pH 8.0, 125 mm NaCl, 2 mm EDTA, inhibitor cocktail [#P1010; Beyotime]). The pellet was collected and resuspended in 200 μL water. Then to the resuspended solution was added 200 μL 25%TCA and incubated on ice for 15 min. Then the pellet was collected and added to 1 mL cold acetone. We vortexed the samples and incubated them at −20 °C for 20 min. The pellet was collected by centrifuging and then analyzed by western blot.

### Statistical analysis

spss v. 20.0 software (IBM, Armonk, NY, USA) was used for the statistical analysis. The results were expressed as the mean ± standard deviation (SD). Studen’'s *t*‐test was used to estimate the significant differences between groups. In all experiments, *P* < 0.05 was considered statistically significant.

## Results

### SETDB2 is required for accurate chromosome segregation and mitosis exiting

**Fig. 1:**
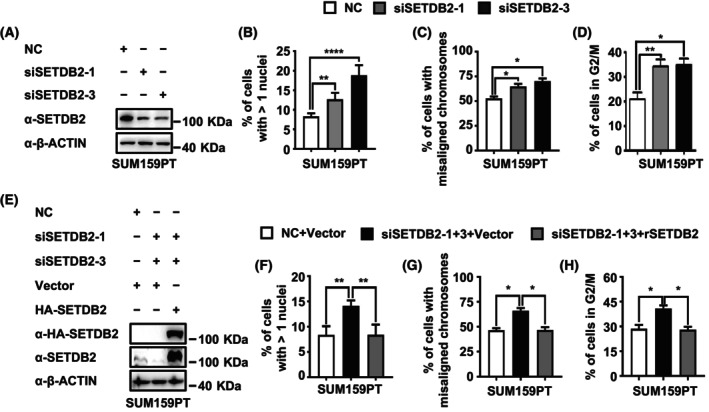
SETDB2 induces accurate chromosome segregation and mitosis exiting. (A,E) Antibodies SETDB2 and HA were used for western blot to analyze (A) SETDB2 knockdown efficiency and (E) SETDB2 expression level in SUM159PT cells. Antibody β‐ACTIN was used as a loading control. (B,F) The percentage of cells with >1 nuclei in (B) SETDB2 knockdown and (F) SETDB2 rescued SUM159PT cells were quantified. Cells were stained with antibody β‐TUBULIN. Nuclei were stained with DAPI. The cell numbers (shown in Tables S5 and S6) were over 400 for each group. Data are presented as mean ± SD of three independent experiments. (C,G) Percentage of cells with misaligned chromosomes in (C) SETDB2 knockdown and (G) SETDB2 rescued SUM159PT cells were quantified. Spindles were stained with antibody β‐TUBULIN. Chromosomes were stained with DAPI. Data are presented as mean ± SD of three independent experiments. (D) SETDB2 knockdown and (H) SETDB2 rescue SUM159PT cells were synchronized and released the cell cycle for 4 h, then the cells were fixed and analyzed by flow cytometry and the percentages of cells in G2/M phase were quantified. Student's *t*‐test was used to estimate the significant differences between groups in (B,C,D,F,G,H). Data are presented as mean ± SD of three independent experiments. ****P < 0.001, **P < 0.01 and *P < 0.05.

It is reported that SETDB2 depletion delays mitotic progression from prophase to anaphase and associates with chromosomal abnormalities [2]. In order to confirm the function of SETDB2 in cell mitotic progression, we knocked down SETDB2 (Fig. 1A) in SUM159PT breast cancer cells. We found that SETDB2 knockdown significantly increased the percentage of abnormal nuclei in SUM159PT breast cancer cells (Fig. S1A and Fig. 1B), abnormal spindle and chromosome segregation in metaphase and anaphase of mitosis (Fig. S1B and Fig. 1C), and G2/M arrested cells in SUM159PT breast cancer cells (Fig. S1C and Fig. 1D). Restoring SETDB2 in SETDB2 knockdown SUM159PT breast cancer cells (Fig. 1E) significantly decreased the percentage of abnormal nuclei in SUM159PT cells (Fig. S2A and Fig. 1F), rescued the abnormal phenotype of spindle and chromosome segregation in metaphase and anaphase (Fig. S2B and Fig. 1G), and decreased the percentage of G2/M arrested cell in SUM159PT breast cancer cells (Fig. S2C and Fig. 1H). Therefore, all these data indicate that SETDB2 is required for accurate chromosome segregation and mitosis exiting.

### SETDB2 induces accurate chromosome segregation and mitosis exiting in a histone methyltransferase independent way

**Fig. 2:**
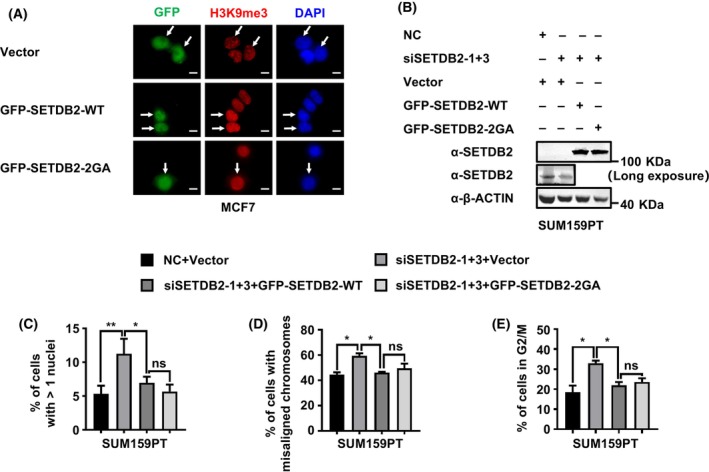
SETDB2 induces accurate chromosome segregation and mitosis exiting independent of its histone methyltransferase activity. (A) Antibody H3K9me3 immunofluorescence staining to analyze the SETDB2 histone methyltransferase activity in SETDB2 wildtype (GFP‐SETDB2‐WT) and SETDB2 histone methyltransferase activity mutation (GFP‐SETDB2‐2GA) overexpression MCF7 cells. The H3K9me3 level is shown by antibody H3K9me3 staining. Nuclei were stained with DAPI. Scale bar: 10 μm. (B) Antibody SETDB2 was used for western blot to analyze SETDB2 knockdown efficiency, SETDB2 wildtype (GFP‐SETDB2‐WT), and SETDB2 histone methyltransferase activity mutation (GFP‐SETDB2‐2GA) expression level in SUM159PT cells. Antibody β‐ACTIN was used as the loading control. (C) The percentage of cells with >1 nuclei in NC + Vector, SETDB2 knockdown + Vector, SETDB2 wildtype (GFP‐SETDB2‐WT), and SETDB2 histone methyltransferase activity mutation (GFP‐SETDB2‐2GA) rescued SUM159PT cells were quantified. Cells were stained with antibody β‐TUBULIN. Nuclei were stained with DAPI. The cell numbers (shown in Table S7) were over 200 for each group. Data are presented as mean ± SD of three independent experiments. (D) The percentage of cells with misaligned chromosomes in NC + Vector, SETDB2 knockdown + Vector, SETDB2 wildtype (GFP‐SETDB2‐WT), and SETDB2 histone methyltransferase activity mutation (GFP‐SETDB2‐2GA) rescued SUM159PT cells were quantified. Spindles were stained with anti‐β‐TUBULIN. Chromosomes were stained with DAPI. (E) NC + Vector, SETDB2 knockdown + Vector and SETDB2 wildtype (GFP‐SETDB2‐WT) and SETDB2 histone methyltransferase activity mutation (GFP‐SETDB2‐2GA) rescued SUM159PT cells were synchronized and released in the cell cycle for 4 h, then the cells were fixed and analyzed by flow cytometry and the percentages of cells in G2/M phase were quantified. Student's *t*‐test was used to estimate the significant differences between groups (C,D,E). Data are presented as mean ± SD of three independent experiments.**P < 0.01 and *P < 0.05; ns, not significant.

It is reported that the function of SETDB2 regulating mitosis and accurate chromosome segregation is associated with H3K9me3 [2]. The Ado‐Met binding motif GxG is conserved among SET family proteins, which are involved in methyl donor AdoMet binding during the methylation reaction [23, 24]. We aligned the Ado‐Met binding motif of SETDB2 and another SET family and found that Ado‐Met binding motif GxG is also conserved in SETDB2 proteins (Fig. S3). Thus, we constructed the SETDB2 Ado‐Met binding mutation 2GA to exasmine whether the functions of accurate chromosome segregation and mitosis exiting were dependent on SETDB2 histone methyltransferase activity. First, we overexpressed SETDB2 wildtype and SETDB2 2GA mutation in MCF7 breast cancer cells, and examined histone methyltransferase activity through H3K9me3 immunofluorescence staining. In SETDB2 wildtype overexpression MCF7 breast cancer cells, the H3K9me3 staining signal significantly increased, while the H3K9me3 staining signal in vector and SETDB2 2GA mutation groups did not change (Fig. 2A). We also examined the H3K9me level in SETDB2 knockdown and GFP‐SETDB2‐WT or GFP‐SETDB2‐2GA rescued MCF7 cells. We found knocking down SETDB2 significantly reduced the globe H3K9me3 level (which is consistent with previous work [2]), restoring GFP‐SETDB2‐WT in SETDB2 knockdown MCF7 cells increased the H3K9me3 level, and restoring GFP‐SETDB2‐2GA did not increase the H3K9me3 level (Fig. S4). These results suggested that SETDB2 is an H3K9me3 methyltransferase and the 2GA mutation is an H3K9me3 methyltransferase function defect mutation. We further restored SETDB2 wildtype and SETDB2 2GA mutation in SETDB2 knockdown SUM159PT breast cancer cells (Fig. 2B and Fig. S6), and found that both SETDB2 wildtype and SETDB2 2GA mutation significantly decreased the percentage of abnormal nuclei (Fig. S5A and Fig. 2C), abnormal phenotype of spindle and chromosome segregation in metaphase and anaphase (Fig. S5B and Fig. 2D), and G2/M arrested cell (Fig. S5C and Fig. 2E). These data indicate that SETDB2 induces accurate chromosome segregation and mitosis exiting in a histone methyltransferase‐independent way.

### SETDB2 interacts with BUBR1, promotes CDC20 to bind to BUBR1 and APC3 and CYCLIN B1 degradation

**Fig. 3:**
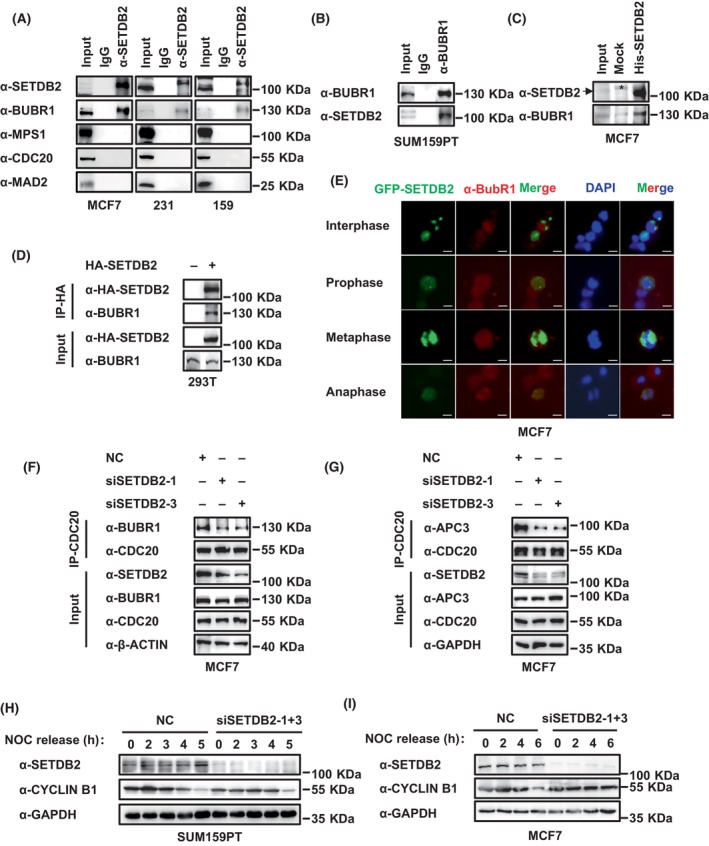
SETDB2 interacts with BUBR1, promotes CDC20 to bind BUBR1 and APC3, and CYCLIN B1 degradation. (A,B) The endogenous interaction between SETDB2 and BUBR1, MPS1, CDC20, or MAD2 in MCF7, MDA‐MB‐231, and SUM159PT cells. (C) His‐tag pulldown showed the interaction between exogenous His‐SETDB2 and endogenous BUBR1 in MCF7 cells. The unspecific band was marked with an asterisk (*). (D) The interaction between exogenous HA‐SETDB2 and endogenous BUBR1 in 293T cells. (E) Localization of GFP‐SETDB2 and BUBR1 in MCF7 cells during interphase, prophase, metaphase, and anaphase (scale bar, 10 μm). Chromosomes were stained with DAPI. Scale bar: 10 μm. (F,G) The endogenous interaction between CDC20 and (F) BUBR1 or (G) APC3 in NC and SETDB2 knockdown MCF7 cells after being synchronized. Antibody SETDB2 showed SETDB2 knockdown efficiency. Antibodies β‐ACTIN or GAPDH were used as the loading control. (H) Western blot for SETDB2 and CYCLIN B1 in NC, and SETDB2 knockdown SUM159PT cells synchronized and released cell cycle for 0, 2, 3, 4, and 5 h. Antibody GAPDH was used as a loading control. (I) Western blot for SETDB2 and CYCLIN B1 in NC, and SETDB2 knockdown MCF7 cells synchronized and released cell cycle for 0, 2, 4, and 6 h. Antibody GAPDH was used as the loading control.

Considering the critical roles of APC/C complex regulation protein in accurate chromosome segregation and mitosis exiting, we examined whether SETDB2 interacted with these proteins. Immunoprecipitation showed that SETDB2 endogenously interacted with BUBR1 rather than MPS1, CDC20, or MAD2 in MCF7, MDA‐MB‐231, and SUM159PT breast cancer cells (Fig. 3A,B). In addition, exogenous SETDB2 interacted with endogenous BUBR1 in HA‐SETDB2 overexpression of 293T cells (Fig. 3D). Pulldown also showed that exogenous His‐SETDB2 expressed by *E. coli* also interacted with endogenous BUBR1 of MCF7 cells (Fig. 3C). In MCF7 cells, the immunofluorescence showed that GFP‐SETDB2 and endogenous BUBR1 were partially colocalized during metaphase, but not during interphase, prophase, or anaphase (Fig. 3E). In addition, we also found that SETDB2 knockdown diminished the interaction between CDC20 and BUBR1 or APC3 (APC/C complex component protein) in MCF7 breast cancer cells (Fig. 3F,G). As a CDC20‐interacting protein, BUBR1 not only interacts with BUB3, MAD2, and CDC20 to form the MCC complex, then binds and inhibits APC/C activation [25, 26], but also acts as a substrate specifier for PP2A‐B56 to dephosphorylate CDC20, leading to CDC20 activation and APC/C binding and activation [22]. Thus, we examined the protein level of CYCLIN B1, whose degradation is considered a result of APC/C^CDC20^ complex activation and a marker of mitosis exiting. In SUM159PT cells, we observed that the CYCLIN B1 level of the NC group was lower than the SETDB2 knockdown group after nocodazole is released at 5 h (Fig. 3H). In MCF7 cells, the CYCLIN B1 protein level of the NC group was lower than the SETDB2 knockdown group after nocodazole is released at 6 h (Fig. 3I). These results indicated that SETDB2 promotes CYCLIN B1 degradation, suggesting that the APC/C complex was deactivated. This result is consistent with the result that SETDB2 knockdown led to abnormal chromosome segregation and G2/M cell arrest. These data indicate that SETDB2 interacts with BUBR1, promotes CDC20 to bind BUBR1 and APC3, and promotes CYCLIN B1 degradation.

### SETDB2 interacts with BUBR1, promotes CDC20 binding to BUBR1 and APC3, and promotes CYCLIN B1 degradation independently of its histone methyltransferase activity

**Fig. 4:**
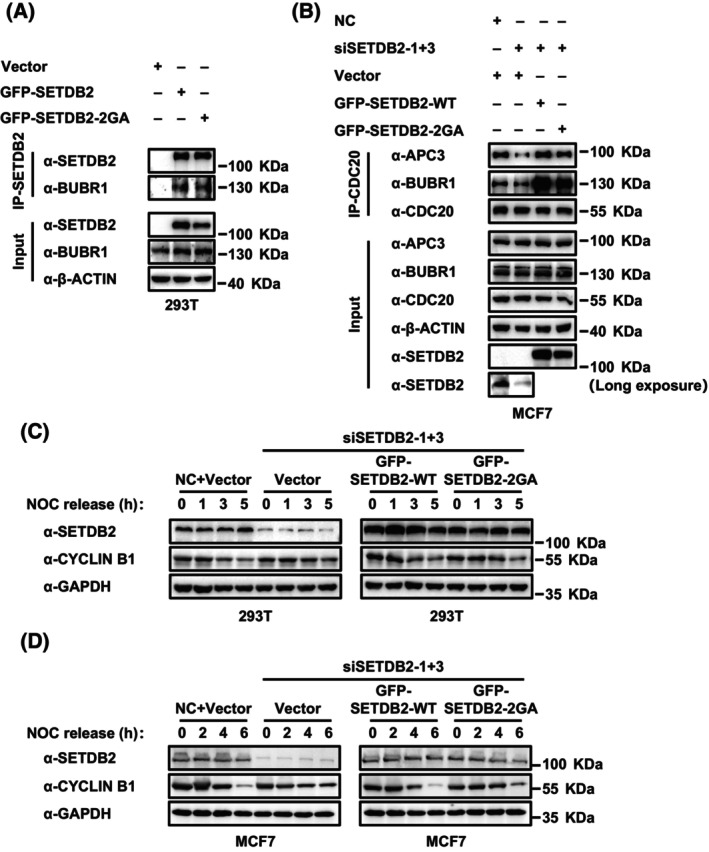
SETDB2 interacts with BUBR1, promotes CDC20 to bind BUBR1 and APC3, and CYCLIN B1 degradation in histone methyltransferase activity in an independent way. (A) The interaction between exogenous GFP‐SETDB2‐WT or GFP‐SETDB2‐2GA and endogenous BUBR1 in 293T cells. Antibody β‐ACTIN was used as a loading control. (B) The endogenous interaction between CDC20 and BUBR1 or APC3 in SETDB2 knockdown, SETDB2 wildtype (GFP‐SETDB2‐WT), and SETDB2 histone methyltransferase activity mutation (GFP‐SETDB2‐2GA) rescued MCF7 cells after being synchronized. Antibody SETDB2 showed the SETDB2 level. Antibody β‐ACTIN was used as a loading control. (C) Western blot for SETDB2 and CYCLIN B1 in NC + Vector, SETDB2 knockdown + Vector, SETDB2 wildtype (GFP‐SETDB2‐WT), and SETDB2 histone methyltransferase activity mutation (GFP‐SETDB2‐2GA) rescued 293T cells synchronized and released in the cell cycle for 0, 1, 3 and 5 h. Antibody GAPDH was used as a loading control. (D) Western blot for SETDB2 and CYCLIN B1 in NC + Vector, SETDB2 knockdown + Vector, SETDB2 wildtype (GFP‐SETDB2‐WT), and SETDB2 histone methyltransferase activity mutation (GFP‐SETDB2‐2GA) rescued MCF7 cells synchronized and released cell cycle for 0, 2, 4 and 6 h. Antibody GAPDH was used as the loading control.

We further examined whether the histone methyltransferase activity of SETDB2 was involved in the interaction between SETDB2 and BUBR1. We found that exogenous SETDB2 wildtype and SETDB2 2GA mutation showed the same binding ability on endogenous BUBR1 in 293T cells (Fig. 4A). Restoring both SETDB2 wildtype and SETDB2 2GA mutation in SETDB2 knockdown MCF cells rescued the binding ability of CDC20 on BUBR1 and APC3 (Fig. 4B). We also examined the protein level of CYCLIN B1 in SETDB2 knockdown, SETDB2 wildtype, and SETDB2 2GA mutation rescued cells. In 293T cells, we observed that the CYCLIN B1 level of the NC + Vector group was lower than the SETDB2 knockdown + Vector group after nocodazole released 5 h (Fig. 4C), and both restoring SETDB2 wildtype and SETDB2 2GA mutation in SETDB2 knockdown cells significantly reduced the CYCLIN B1 level compared to the NC + Vector group after nocodazole released 5 h (Fig. 4C). In MCF7 cells, we also found the CYCLIN B1 level the of NC + Vector group was lower than the SETDB2 knockdown + Vector group after nocodazole released 6 h (Fig. 4D), and both restoring the SETDB2 wildtype and SETDB2 2GA mutation in SETDB2 knockdown cells significantly reduced the CYCLIN B1 level compared the to NC + Vector group after nocodazole released 6 h (Fig. 4D). These data indicate that SETDB2 interacts with BUBR1 and promotes CDC20 to bind to BUBR1 and APC3 and CYCLIN B1 degradation in a histone methyltransferase activity in an independent way.

In conclusion, histone methyltransferase SETDB2 interacts with BUBR1, promotes CDC20 to bind BUBR1 and APC3, then degrades CYCLIN B1 to guarantee accurate chromosome segregation and mitosis exiting in a histone methyltransferase activity in an independent way.

## Discussion

It is reported that the function of SETDB2 regulating mitosis and accurate chromosome segregation is associated with H3K9me3 [2]. However, in our study we found the functions of SETDB2 on regulating mitosis and chromosome accurate segregation are independent of its histone methyltransferase activity. The mechanism analysis shows that SETDB2 interacts with BUBR1 to promote CDC20 to bind BUBR1 and APC3, then degrades CYCLIN B1 to guarantee accurate chromosome segregation and mitosis exiting in a histone methyltransferase activity in an independent way.

It is reported that SETDB2 participates in chromosome condensation and segregation, and SETDB2 knockdown delays mitosis. This function is associated with H3K9me3 distribution on repetitive DNA, HP1 recruitment, and CENP proteins location [2]. The phenotype of abnormal chromosome segregation and delayed mitosis in the SETDB2 depleted cell is consistent with our observation. However, whether SETDB2 induced accurate chromosome segregation and mitosis exiting dependent on its histone methyltransferase activity still lacks provable direct evidence. In this study, we used SETDB2 2GA mutation (histone methyltransferase activity mutation) to examine whether the functions of accurate chromosome segregation and mitosis exiting induced by SETDB2 are dependent on its histone methyltransferase activity. Both SETDB2 wildtype and SETDB2 2GA mutation rescued the phenotype of abnormal chromosome segregation and delayed mitosis, suggesting SETDB2 induced accurate chromosome segregation and mitosis exiting in a histone methyltransferase activityin an independent way.

CENP‐A is an essential histone variant that replaces the canonical H3 at the centromeres and marks these regions epigenetically [27]. CENP‐C is a key factor that binds to CENP‐A nucleosomes and stabilizes centromeric chromatin to promote CENP‐A chromatin assembly [28, 29, 30]. CENP‐B also interacts with CENP‐A nucleosomes and CENP‐C to stabilize the CENP‐A nucleosome and maintain kinetochore function [31, 32]. In Falandry *et al.'s* work, SETDB2 depletion coincides with CENP‐B and CENP‐C loss and mitosis delay [2], suggesting that SETDB2 may play important roles in CENP‐A chromatin assembly. In addition, CENP‐A‐depleted cells exhibit a specific defect in maintaining kinetochore localization of BubR1 under conditions of checkpoint activation [33]. The mitotic delay caused by null mutations of CENP‐A can be partially suppressed by BUBR1 mutation, suggesting that checkpoint activation can occur through a BUBR1‐dependent pathway [33, 34]. In our study we found that SETDB2 interacted with BUBR1 and implied that there may be a crosstalk among BUBR1, SETDB2, and CENP‐A chromatin assembly during mitosis. The regulation mechanism awaits further exploration.

Taken together, histone methyltransferase SETDB2 induces accurate chromosome segregation and mitosis through interacting with BUBR1 in histone methyltransferase activity in an independent way. Our study reveals a novel mechanism of SETDB2 regulating accurate chromosome segregation and mitosis exiting.

## Conflict of interest

The authors declare no conflicts of interest.

### Peer review

The peer review history for this article is available at https://www.webofscience.com/api/gateway/wos/peerreview/10.1002/2211-5463.13761.

## Author contributions

JL and XM were involved in study design; YT, HZ, JX, YZ, RY, and JF were involved in study conduct, data analysis, and its interpretation; JL and YT were involved in article drafting.

## Supporting information


**Fig. S1.** SETDB2 expression is required for accurate chromosome segregation and proper mitosis.
**Fig. S2.** Restored SETDB2 level rescues abnormal chromosome segregation and mitosis defects.
**Fig. S3.** The protein sequence alignment of the Ado‐Met binding motif in SET family protein.
**Fig. S4.** SETDB2 did not change the globe H3K9me3 level.
**Fig. S5.** SETDB2 induces accurate chromosome segregation and proper mitosis in histone methyltransferase activity independent way.
**Fig. S6.** The whole panel of immunoblots in Fig. 2B.

**Table S1.** The sequence of SETDB2 in pEGFP‐SETDB2‐WT plasmid.
**Table S2.** The sequence of SETDB2 in pEGFP‐SETDB2‐2GA plasmid.
**Table S3.** The primer sequences used for Site‐Directed Mutagenesis.
**Table S4.** The sequences of siRNAs.

**Table S5.** The cell count and the percentage of abnormal nuclear cell in Fig. 1B.
**Table S6.** The cell count and the percentage of abnormal nuclear cell in Fig. 1F.
**Table S7.** The cell count and the percentage of abnormal nuclear cell in Fig. 2C.

## Data Availability

The data that support the findings of this study are available in the Supporting Information of this article.
