# Supplementary material for: SETDB2 interacts with BUBR1 to induce accurate chromosome segregation independently of its histone methyltransferase activity
Source: FEBS Open Bio. 2024 Jan 9;14(3):444–54. doi: 10.1002/2211-5463.13761 (PMC10909981; doi:10.1002/2211-5463.13761)
Supplement: Supplementary file 1 — Fig. S1. SETDB2 expression is required for accurate chromosome segregation and proper mitosis. Fig. S2. Restored SETDB2 level rescues abnormal chromosome segregation and mitosis defects. Fig. S3. The protein sequence alignment of the Ado‐Met binding motif in SET family protein. Fig. S4. SETDB2 did not change the globe H3K9me3 level. Fig. S5. SETDB2 induces accurate chromosome segregation and proper mitosis in histone methyltransferase activity independent way. Fig. S6. The whole panel of immunoblots in Fig. 2B. [file FEB4-14-444-s002.zip › feb413761-sup-0001-FigsS1-S6.pdf]

Fig.S1

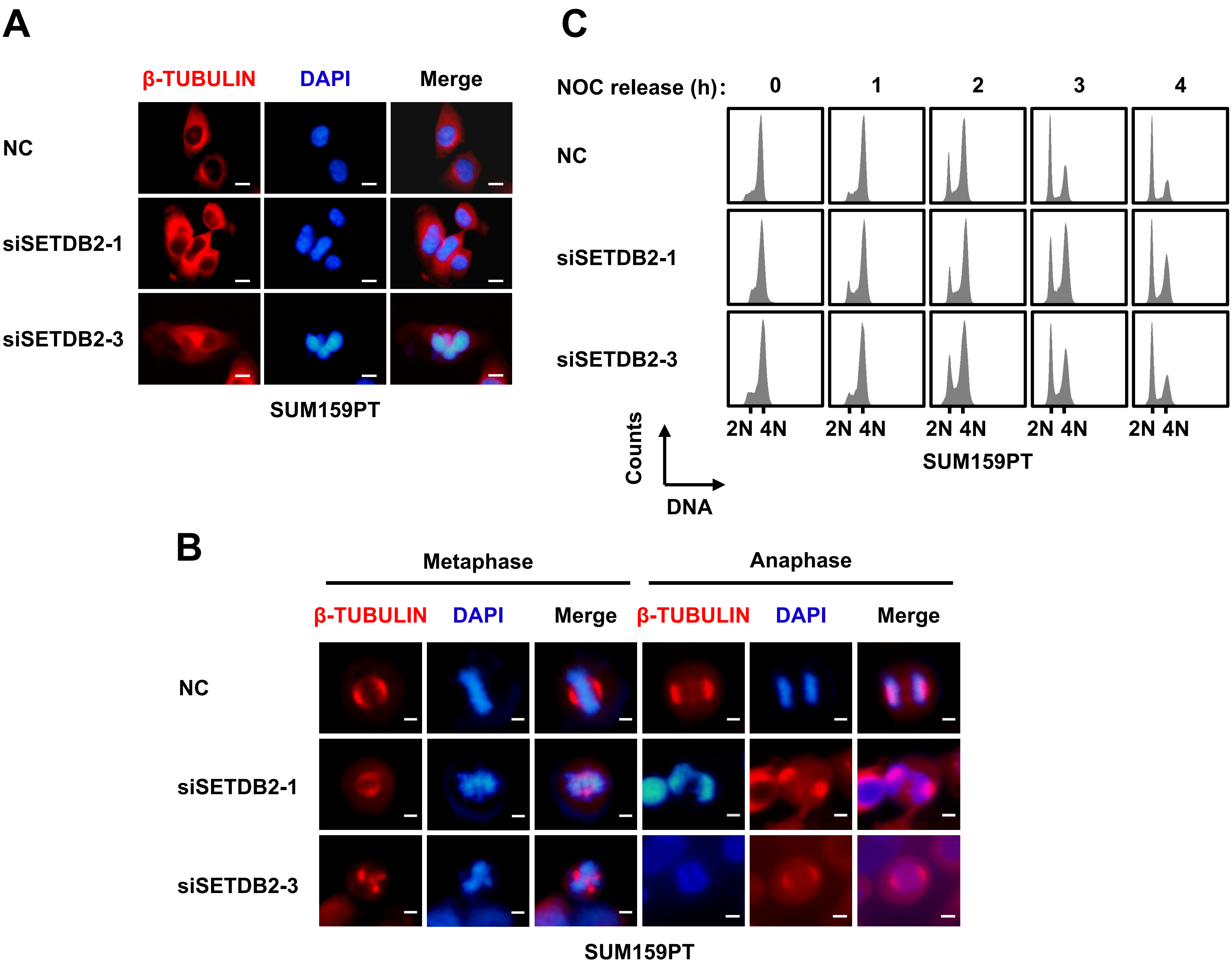
A
β-TUBULIN
DAPI
Merge
NC
siSETDB2-1
siSETDB2-3
SUM159PT
C
NOC release (h):
0
1
2
3
4
NC
siSETDB2-1
siSETDB2-3
2N 4N
Counts
DNA
SUM159PT
B
Metaphase
Anaphase
β-TUBULIN
DAPI
Merge
NC
siSETDB2-1
siSETDB2-3
SUM159PT

Fig.S2

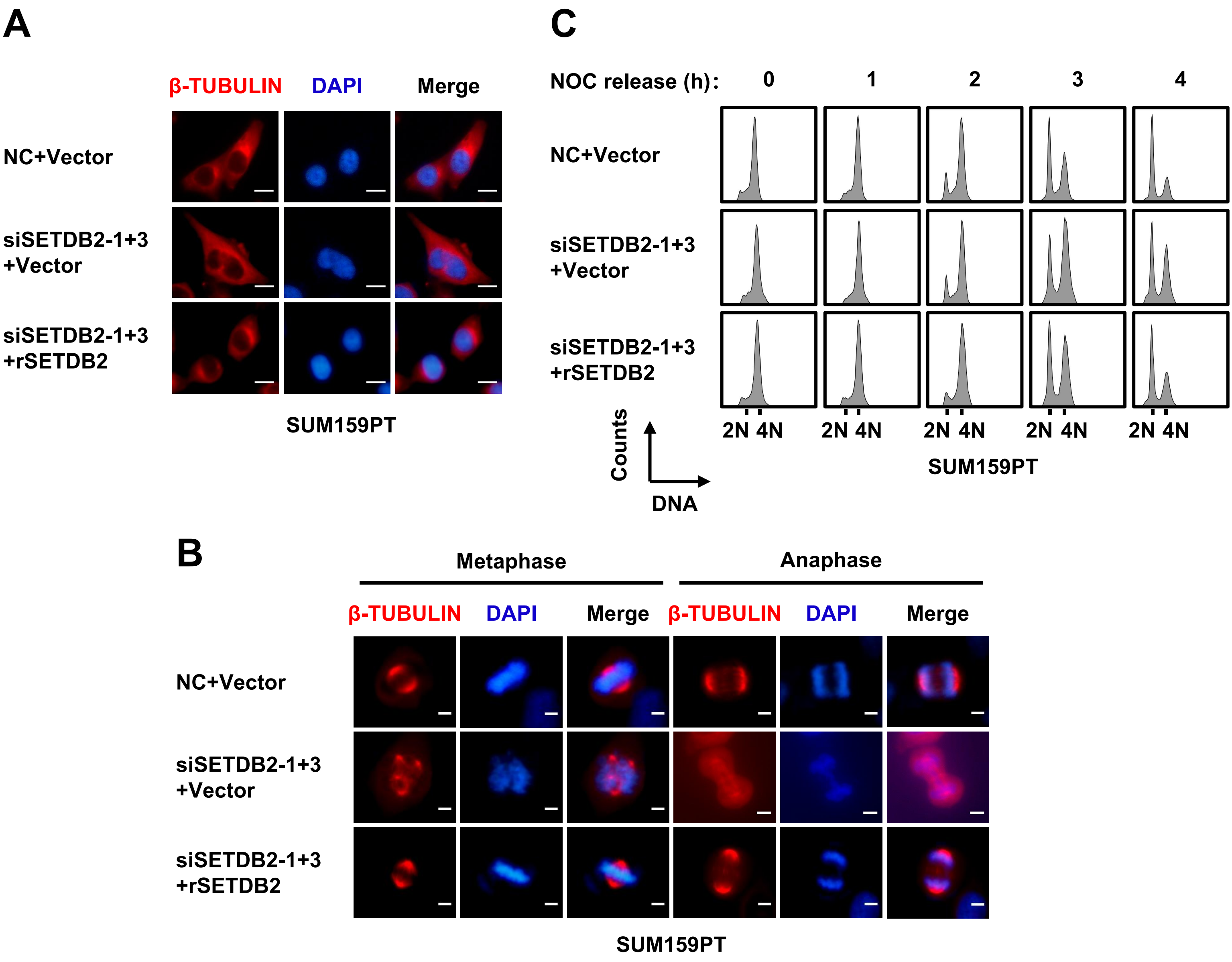
A
β-TUBULIN
DAPI
Merge
NC+Vector
siSETDB2-1+3
+Vector
siSETDB2-1+3
+rSETDB2
SUM159PT
C
NOC release (h):
0
1
2
3
4
NC+Vector
siSETDB2-1+3
+Vector
siSETDB2-1+3
+rSETDB2
Counts
DNA
2N 4N
2N 4N
2N 4N
2N 4N
2N 4N
SUM159PT
B
Metaphase
Anaphase
β-TUBULIN
DAPI
Merge
β-TUBULIN
DAPI
Merge
NC+Vector
siSETDB2-1+3
+Vector
siSETDB2-1+3
+rSETDB2
SUM159PT

Fig.S3

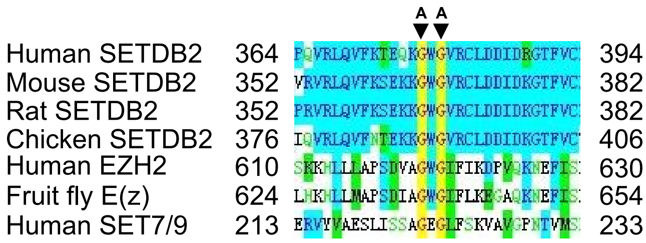
A A
Human SETDB2 364 PQVRLQVFKTEQKGWGVRCLDDIDRGTFVC 394
Mouse SETDB2 352 VRVRLQVFKSEKKGWGVRCLDDIDKGTFVC 382
Rat SETDB2 352 PRVRLQVFKSEKKGWGVRCLDDIDKGTFVC 382
Chicken SETDB2 376 IQVRLQVFNTEKKGWGVRCLDDIDKGTFVC 406
Human EZH2 610 SKKHLLLAPSDVAGWGIFIKDPVQKNEFIS 630
Fruit fly E(z) 624 LHKHLLMAPSDIAGWGIFLKEGAQKNEFIS 654
Human SET7/9 213 ERVYVAESLISSAGEGLFSKVAVGPNTVMS 233

Fig.S4

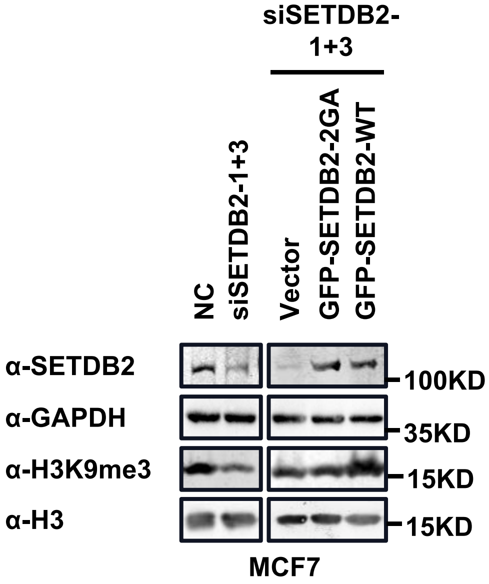
siSETDB2-
1+3
NC
siSETDB2-1+3
Vector
GFP-SETDB2-2GA
GFP-SETDB2-WT
α-SETDB2
α-GAPDH
α-H3K9me3
α-H3
100KD
35KD
15KD
15KD
MCF7

Fig.S5

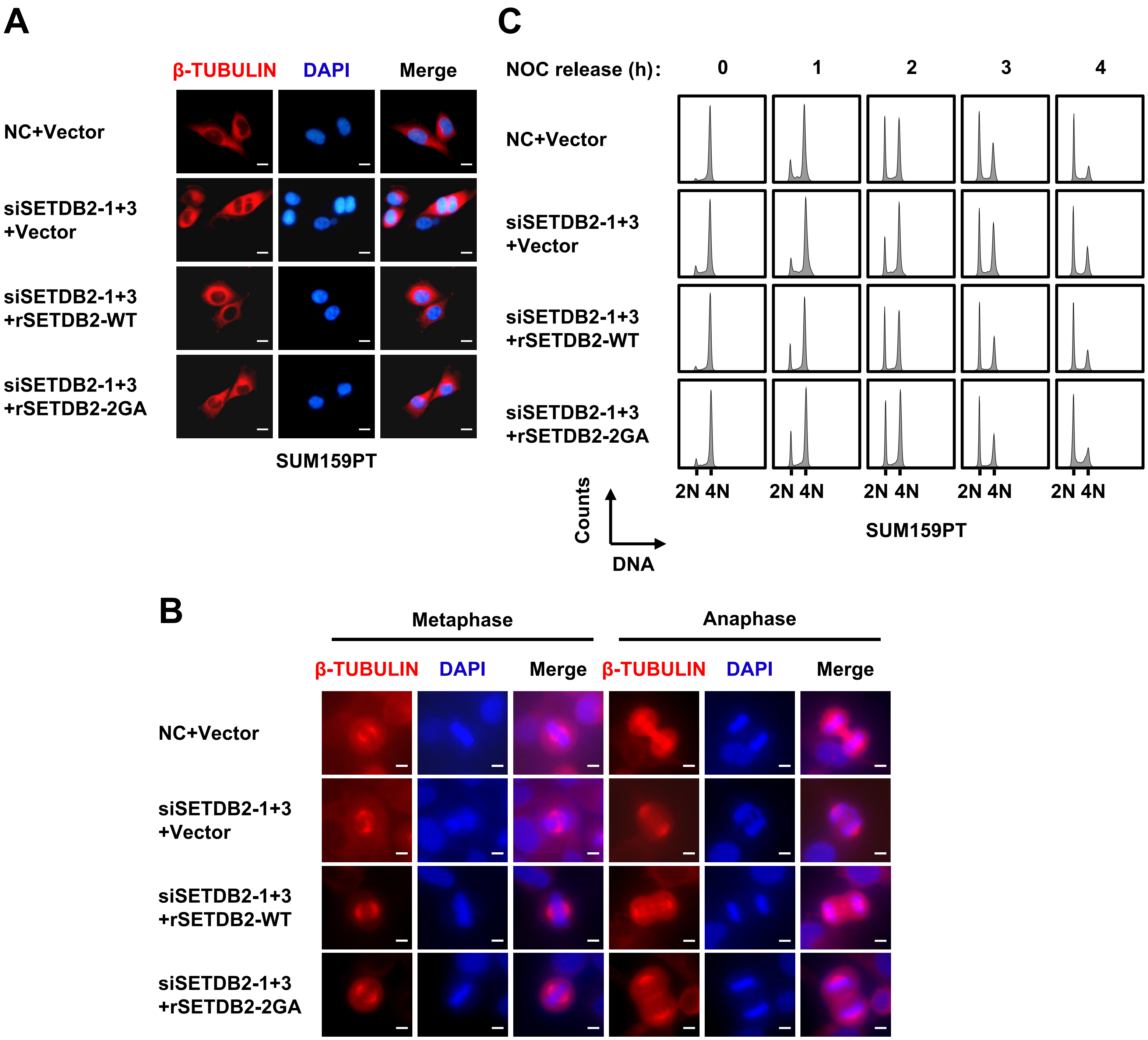
A
β-TUBULIN
DAPI
Merge
NC+Vector
siSETDB2-1+3
+Vector
siSETDB2-1+3
+rSETDB2-WT
siSETDB2-1+3
+rSETDB2-2GA
SUM159PT
C
NOC release (h):
0
1
2
3
4
NC+Vector
siSETDB2-1+3
+Vector
siSETDB2-1+3
+rSETDB2-WT
siSETDB2-1+3
+rSETDB2-2GA
2N 4N
Counts
DNA
SUM159PT
B
Metaphase
Anaphase
β-TUBULIN
DAPI
Merge
NC+Vector
siSETDB2-1+3
+Vector
siSETDB2-1+3
+rSETDB2-WT
siSETDB2-1+3
+rSETDB2-2GA

Fig.S6

A

NC	+	-	-	-
siSETDB2-1+3	-	+	+	+
Vector	+	+	-	-
GFP-SETDB2-WT	-	-	+	-
GFP-SETDB2-2GA	-	-	-	+

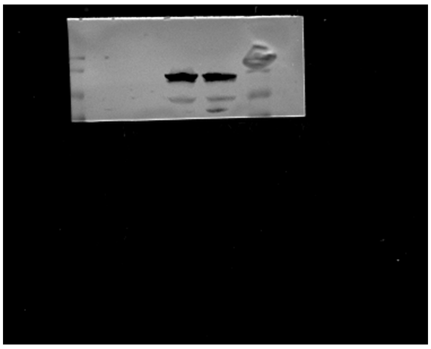

short exposure

B

NC	+	-	-	-
siSETDB2-1+3	-	+	+	+
Vector	+	+	-	-
GFP-SETDB2-WT	-	-	+	-
GFP-SETDB2-2GA	-	-	-	+

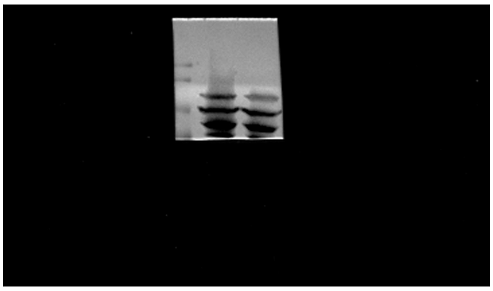

Long exposure
