# Supplementary material for: SETDB2 interacts with BUBR1 to induce accurate chromosome segregation independently of its histone methyltransferase activity
Source: FEBS Open Bio. 2024 Jan 9;14(3):444–54. doi: 10.1002/2211-5463.13761 (PMC10909981; doi:10.1002/2211-5463.13761)
Supplement: Supplementary file 1 — Fig. S1. SETDB2 expression is required for accurate chromosome segregation and proper mitosis. Fig. S2. Restored SETDB2 level rescues abnormal chromosome segregation and mitosis defects. Fig. S3. The protein sequence alignment of the Ado‐Met binding motif in SET family protein. Fig. S4. SETDB2 did not change the globe H3K9me3 level. Fig. S5. SETDB2 induces accurate chromosome segregation and proper mitosis in histone methyltransferase activity independent way. Fig. S6. The whole panel of immunoblots in Fig. 2B. [file FEB4-14-444-s002.zip › Figure_Legends.docx]

**SUPPLEMENTARY FIGURE LEGENDS**

**Fig. S1. SETDB2 expression is required for accurate chromosome segregation and proper mitosis. (A)** Representative microphotographs for NC and SETDB2 knockdown SUM159PT cells which were stained with antibody β-TUBULIN (scale bar, 10μm). Nuclei were stained with DAPI. **(B)** Representative microphotographs for NC and SETDB2 knockdown SUM159PT cells during metaphase and anaphase (scale bar, 5μm). The spindles were stained with antibody β-TUBULIN. Chormosomes were stained with DAPI. **(C)** NC and SETDB2 knockdown SUM159PT cells were synchronized and released the cell cycle for 0, 1, 2, 3, and 4 hours, then the cells were fixed and analyzed by flow cytometry. The positions of 2N (G1) and 4N (G2/M) DNA contents were indicated.

**Fig. S2. Restored SETDB2 level rescues abnormal chromosome segregation and mitosis defects. (A)** Representative microphotographs for cells of NC, SETDB2 knockdown and rescued SUM159PT cells (scale bar,10μm) which were stained with antibody β-TUBULIN. Nuclei were stained with DAPI. **(B)** Representative microphotographs for NC, SETDB2 knockdown and rescued SUM159PT cells during metaphase and anaphase (scale bar, 5μm). The spindles were stained with antibody β-TUBULIN. Chormosomes were stained with DAPI. **(C)** NC, SETDB2 knockdown and rescued SUM159PT cells were synchronized and released the cell cycle for 0, 1, 2, 3, and 4 hours, then the cells were fixed and analyzed by flow cytometry. The positions of 2N (G1) and 4N (G2/M) DNA contents were indicated.

**Fig. S3. The protein sequence alignment of the Ado-Met binding motif in SET family protein.** The Ado-Met binding motif sequence were aligned among human SETDB2, mouse SETDB2, rat SETDB2, chicken SETDB2, human EZH2, fruit fly E(z) (which is a homologous protein of human EZH2), human SET7/9 proteins. The arrow marks the conserved Glycine of GxG Ado-Met binding motif.

**Fig. S4. SETDB2 did not change the globe H3K9me3 level.** Antibodies against SETDB2 were used for Western blot to analyze SETDB2 knockdown efficiency in MCF7 cells. Antibody GAPDH was used as loading control.

**Fig. S5. SETDB2 induces accurate chromosome segregation and proper mitosis in histone methyltransferase activity independent way. (A)** Representative microphotographs for cells of NC, SETDB2 knockdown, SETDB2 wildtype (GFP-SETDB2-WT) and SETDB2 histone methyltransferas activity mutation (GFP-SETDB2-2GA) rescued SUM159PT cells (scale bar,10μm). These cells were stained with antibody β-TUBULIN. Nuclei were stained with DAPI. **(B)** Representative microphotographs for NC, SETDB2 knockdown, SETDB2 wildtype (GFP-SETDB2-WT) and SETDB2 histone methyltransferas activity mutation (GFP-SETDB2-2GA) rescued SUM159PT cells during metaphase and anaphase (scale bar, 5μm). The spindles were stained with antibody β-TUBULIN. Chormosomes were stained with DAPI. **(C)** NC, SETDB2 knockdown, SETDB2 wildtype (GFP-SETDB2-WT) and SETDB2 histone methyltransferas activity mutation (GFP-SETDB2-2GA) rescued SUM159PT cells were synchronized and released the cell cycle for 0, 1, 2, 3, and 4 hours, then the cells were fixed and analyzed by flow cytometry. The positions of 2N (G1) and 4N (G2/M) DNA contents were indicated.

**Fig. S6. The whole panel of immunoblots in Fig.2B. (A)** Antibody SETDB2 was used for Western blot to analyze SETDB2 wildtype (GFP-SETDB2-WT) and SETDB2 histone methyltransferase activity mutation (GFP-SETDB2-2GA) expression level in SUM159PT cells. The NC member were exposed for a short time. **(B)** Antibody SETDB2 was used for Western blot to analyze SETDB2 knockdown efficiency in SUM159PT cells. The NC member were exposed for a long time.
