# Supplementary material for: SETDB2 interacts with BUBR1 to induce accurate chromosome segregation independently of its histone methyltransferase activity
Source: FEBS Open Bio. 2024 Jan 9;14(3):444–54. doi: 10.1002/2211-5463.13761 (PMC10909981; doi:10.1002/2211-5463.13761)
Supplement: Supplementary file 2 — Table S1. The sequence of SETDB2 in pEGFP‐SETDB2‐WT plasmid. Table S2. The sequence of SETDB2 in pEGFP‐SETDB2‐2GA plasmid. Table S3. The primer sequences used for Site‐Directed Mutagenesis. Table S4. The sequences of siRNAs. [file FEB4-14-444-s001.docx]

## Table S1 The sequence of SETDB2 in pEGFP-SETDB2-WT plasmid.

| ATGTATCCTTACGACGTGCCTGACTACGCCGGAGAGAAGAACGGCGACGCCAAGACCTTTTGGATGGAGCTCGAGGACGACGGCAAGGTGGACTTCATCTTCGAGCAGGTGCAGAACGTGCTGCAGAGCCTGAAGCAGAAGATCAAGGACGGCAGCGCCACCAACAAGGAGTACATCCAGGCCATGATCCTGGTGAACGAGGCCACAATCATCAACAGCAGCACCAGCATCAAGGGCGCCTCTCAGAAGGAAGTGAACGCCCAGAGCAGCGACCCTATGCCAGTGACCCAGAAGGAGCAGGAGAACAAGAGCAACGCCTTCCCCAGCACCTCTTGCGAGAACAGCTTCCCCGAGGACTGCACCTTCCTGACCACCGAGAACAAGGAGATCCTGAGCCTGGAGGACAAGGTGGTGGACTTCCGGGAGAAGGACAGCAGCAGCAACCTGAGCTACCAGAGCCACGATTGTAGCGGCGCTTGCCTGATGAAGATGCCCCTGAACCTGAAGGGAGAGAACCCTCTGCAGCTGCCCATCAAGTGCCACTTCCAGAGGAGGCACGCCAAGACAAACTCTCACAGCAGCGCCCTGCACGTGTCTTACAAGACCCCTTGCGGCAGGAGCCTGAGAAACGTGGAGGAGGTGTTCCGCTACCTGCTGGAGACCGAGTGCAACTTCCTGTTCACCGACAACTTCAGCTTCAACACCTACGTGCAGCTGGCCAGGAACTACCCCAAGCAGAAGGAGGTCGTGTCCGACGTGGACATCAGCAACGGAGTGGAGAGCGTGCCCATCAGCTTCTGCAACGAGATCGACAGCCGGAAGCTGCCCCAGTTCAAGTACCGGAAAACCGTCTGGCCCAGAGCCTACAACCTGACCAACTTCAGCAGCATGTTCACCGACTCTTGCGACTGCTCCGAGGGGTGCATTGACATCACCAAGTGCGCTTGCCTGCAGCTGACCGCTAGAAACGCCAAGACCAGCCCTCTGAGCAGCGACAAGATCACCACCGGCTACAAGTACAAGCGGCTGCAGAGGCAGATCCCCACAGGCATCTACGAGTGCAGCCTCCTGTGCAAATGCAACCGCCAGCTCTGCCAGAACAGAGTGGTGCAGCACGGACCTCAGGTCCGACTGCAGGTGTTCAAGACCGAGCAGAAGGGTTGGGGCGTGAGATGTCTGGACGACATCGACAGGGGCACCTTCGTCTGCATCTACTCCGGCAGGCTGCTGTCTCGGGCCAACACCGAGAAGAGCTACGGAATCGACGAGAACGGCAGGGACGAGAACACCATGAAGAACATCTTCAGCAAGAAGCGGAAGCTGGAGGTGGCTTGCAGCGATTGCGAGGTGGAAGTGCTGCCACTGGGACTGGAAACCCACCCTAGAACCGCCAAGACCGAGAAGTGCCCCCCCAAGTTCAGCAACAACCCCAAGGAGCTGACCGTGGAGACCAAGTACGACAACATCAGCCGGATCCAGTACCACAGCGTGATCAGGGACCCAGAGAGCAAGACCGCCATCTTCCAGCACAACGGCAAGAAGATGGAGTTCGTGTCCAGCGAGAGCGTGACACCAGAGGACAACGACGGCTTCAAGCCTCCTAGGGAGCACCTGAACAGCAAGACCAAGGGAGCCCAGAAGGACAGCAGCAGCAACCACGTGGACGAGTTCGAGGACAACCTGCTGATCGAGAGCGACGTGATCGACATCACCAAGTACCGGGAGGAGACCCCTCCTAGAAGTCGCTGTAACCAGGCCACCACCCTGGACAACCAGAACATCAAGAAGGCCATCGAGGTGCAGATCCAGAAGCCCCAGGAGGGAAGAAGCACAGCTTGCCAGAGGCAGCAGGTCTTCTGCGACGAAGAGCTGCTGAGCGAGACCAAGAACACCAGCAGCGACAGCCTGACCAAGTTCAACAAGGGCAACGTGTTCCTGCTGGACGCCACAAAGGAGGGAAACGTGGGCCGGTTCCTGAACCACTCTTGTTGCCCCAACCTGCTGGTGCAGAACGTGTTCGTGGAGACCCACAACCGGAACTTCCCTCTGGTGGCCTTCTTCACCAACCGCTACGTGAAGGCCAGAACCGAGCTGACTTGGGACTACGGCTACGAAGCCGGAACAGTGCCCGAGAAGGAGATCTTTTGCCAGTGCGGGGTCAACAAGTGCCGGAAGAAGATCCTG |
| --- |

## Table S2 The sequence of SETDB2 in pEGFP-SETDB2-2GA plasmid.

The 2GA mutant sites were showed in red.

| ATGTATCCTTACGACGTGCCTGACTACGCCGGAGAGAAGAACGGCGACGCCAAGACCTTTTGGATGGAGCTCGAGGACGACGGCAAGGTGGACTTCATCTTCGAGCAGGTGCAGAACGTGCTGCAGAGCCTGAAGCAGAAGATCAAGGACGGCAGCGCCACCAACAAGGAGTACATCCAGGCCATGATCCTGGTGAACGAGGCCACAATCATCAACAGCAGCACCAGCATCAAGGGCGCCTCTCAGAAGGAAGTGAACGCCCAGAGCAGCGACCCTATGCCAGTGACCCAGAAGGAGCAGGAGAACAAGAGCAACGCCTTCCCCAGCACCTCTTGCGAGAACAGCTTCCCCGAGGACTGCACCTTCCTGACCACCGAGAACAAGGAGATCCTGAGCCTGGAGGACAAGGTGGTGGACTTCCGGGAGAAGGACAGCAGCAGCAACCTGAGCTACCAGAGCCACGATTGTAGCGGCGCTTGCCTGATGAAGATGCCCCTGAACCTGAAGGGAGAGAACCCTCTGCAGCTGCCCATCAAGTGCCACTTCCAGAGGAGGCACGCCAAGACAAACTCTCACAGCAGCGCCCTGCACGTGTCTTACAAGACCCCTTGCGGCAGGAGCCTGAGAAACGTGGAGGAGGTGTTCCGCTACCTGCTGGAGACCGAGTGCAACTTCCTGTTCACCGACAACTTCAGCTTCAACACCTACGTGCAGCTGGCCAGGAACTACCCCAAGCAGAAGGAGGTCGTGTCCGACGTGGACATCAGCAACGGAGTGGAGAGCGTGCCCATCAGCTTCTGCAACGAGATCGACAGCCGGAAGCTGCCCCAGTTCAAGTACCGGAAAACCGTCTGGCCCAGAGCCTACAACCTGACCAACTTCAGCAGCATGTTCACCGACTCTTGCGACTGCTCCGAGGGGTGCATTGACATCACCAAGTGCGCTTGCCTGCAGCTGACCGCTAGAAACGCCAAGACCAGCCCTCTGAGCAGCGACAAGATCACCACCGGCTACAAGTACAAGCGGCTGCAGAGGCAGATCCCCACAGGCATCTACGAGTGCAGCCTCCTGTGCAAATGCAACCGCCAGCTCTGCCAGAACAGAGTGGTGCAGCACGGACCTCAGGTCCGACTGCAGGTGTTCAAGACCGAGCAGAAGGCTTGGGCCGTGAGATGTCTGGACGACATCGACAGGGGCACCTTCGTCTGCATCTACTCCGGCAGGCTGCTGTCTCGGGCCAACACCGAGAAGAGCTACGGAATCGACGAGAACGGCAGGGACGAGAACACCATGAAGAACATCTTCAGCAAGAAGCGGAAGCTGGAGGTGGCTTGCAGCGATTGCGAGGTGGAAGTGCTGCCACTGGGACTGGAAACCCACCCTAGAACCGCCAAGACCGAGAAGTGCCCCCCCAAGTTCAGCAACAACCCCAAGGAGCTGACCGTGGAGACCAAGTACGACAACATCAGCCGGATCCAGTACCACAGCGTGATCAGGGACCCAGAGAGCAAGACCGCCATCTTCCAGCACAACGGCAAGAAGATGGAGTTCGTGTCCAGCGAGAGCGTGACACCAGAGGACAACGACGGCTTCAAGCCTCCTAGGGAGCACCTGAACAGCAAGACCAAGGGAGCCCAGAAGGACAGCAGCAGCAACCACGTGGACGAGTTCGAGGACAACCTGCTGATCGAGAGCGACGTGATCGACATCACCAAGTACCGGGAGGAGACCCCTCCTAGAAGTCGCTGTAACCAGGCCACCACCCTGGACAACCAGAACATCAAGAAGGCCATCGAGGTGCAGATCCAGAAGCCCCAGGAGGGAAGAAGCACAGCTTGCCAGAGGCAGCAGGTCTTCTGCGACGAAGAGCTGCTGAGCGAGACCAAGAACACCAGCAGCGACAGCCTGACCAAGTTCAACAAGGGCAACGTGTTCCTGCTGGACGCCACAAAGGAGGGAAACGTGGGCCGGTTCCTGAACCACTCTTGTTGCCCCAACCTGCTGGTGCAGAACGTGTTCGTGGAGACCCACAACCGGAACTTCCCTCTGGTGGCCTTCTTCACCAACCGCTACGTGAAGGCCAGAACCGAGCTGACTTGGGACTACGGCTACGAAGCCGGAACAGTGCCCGAGAAGGAGATCTTTTGCCAGTGCGGGGTCAACAAGTGCCGGAAGAAGATCCTG |
| --- |

## Table S3 The primer sequences used for Site-Directed Mutagenesis

| Primer Name | Sequences (5’-3’) |
| --- | --- |
| SETDB2-2GA-S: | GTGTTCAAGACCGAGCAGAAGGCTTGGGCCGTGAGATGTCTGGACGACATC |
| SETDB2-2GA-AS: | GATGTCGTCCAGACATCTCACGGCCCAAGCCTTCTGCTCGGTCTTGAACAC |

## Table S4 The sequences of siRNAs

| siRNA Name | Sequences (5’-3’) |
| --- | --- |
| siNC | UUCUCCGAACGUGUCACGUTT |
| siSETDB2-1 | GGCGACCACAUUGGAUAAUTT |
| siSETDB2-3 | GGACAUUUGUUUGCAUUUATT |
